# Social Networking Service, Patient-Generated Health Data, and Population Health Informatics: National Cross-sectional Study of Patterns and Implications of Leveraging Digital Technologies to Support Mental Health and Well-being

**DOI:** 10.2196/30898

**Published:** 2022-04-29

**Authors:** Jiancheng Ye, Zidan Wang, Jiarui Hai

**Affiliations:** 1 Feinberg School of Medicine Northwestern University Chicago, IL United States; 2 Department of Statistics, Northwestern University Evanston, IL United States; 3 Department of Hydraulic Engineering, Tsinghua University Beijing China

**Keywords:** patient-generated health data, social network, population health informatics, mental health, social determinants of health, health data sharing, technology acceptability, mobile phone, mobile health

## Abstract

**Background:**

The emerging health technologies and digital services provide effective ways of collecting health information and gathering patient-generated health data (PGHD), which provide a more holistic view of a patient’s health and quality of life over time, increase visibility into a patient’s adherence to a treatment plan or study protocol, and enable timely intervention before a costly care episode.

**Objective:**

Through a national cross-sectional survey in the United States, we aimed to describe and compare the characteristics of populations with and without mental health issues (depression or anxiety disorders), including physical health, sleep, and alcohol use. We also examined the patterns of social networking service use, PGHD, and attitudes toward health information sharing and activities among the participants, which provided nationally representative estimates.

**Methods:**

We drew data from the 2019 Health Information National Trends Survey of the National Cancer Institute. The participants were divided into 2 groups according to mental health status. Then, we described and compared the characteristics of the social determinants of health, health status, sleeping and drinking behaviors, and patterns of social networking service use and health information data sharing between the 2 groups. Multivariable logistic regression models were applied to assess the predictors of mental health. All the analyses were weighted to provide nationally representative estimates.

**Results:**

Participants with mental health issues were significantly more likely to be younger, White, female, and lower-income; have a history of chronic diseases; and be less capable of taking care of their own health. Regarding behavioral health, they slept <6 hours on average, had worse sleep quality, and consumed more alcohol. In addition, they were more likely to visit and share health information on social networking sites, write online diary blogs, participate in online forums or support groups, and watch health-related videos.

**Conclusions:**

This study illustrates that individuals with mental health issues have inequitable social determinants of health, poor physical health, and poor behavioral health. However, they are more likely to use social networking platforms and services, share their health information, and actively engage with PGHD. Leveraging these digital technologies and services could be beneficial for developing tailored and effective strategies for self-monitoring and self-management.

## Introduction

### Background

Mental health issues such as depression and anxiety disorders are severe psychiatric diseases with high prevalence and elevated risks of recurrence and chronicity [1]. There are >260 million people of all ages who have experienced mental illnesses worldwide, which are a leading cause of disability worldwide and a major contributor to the overall global burden of disease [2]. Studies have demonstrated that mental health issues are a strong indicator of poor general health, unhealthy alcohol use, and sleep problems [3,4]. Poor sleep quality has been linked to an increased motivation to drink, especially for young adults [5]. It is critical for patients with mental health issues to receive appropriate health care and social services.

In recent years, there has been increasing acknowledgment of the important role that mental health plays in achieving improved population health. Understanding how these fundamental factors (physical and behavioral health, mental health, and technologies) relate to one another may yield important insights for novel approaches to designing prevention programs and enhancing services for mental health support. Digital health technologies such as smartphone apps and social media provide opportunities to continuously collect objective information on behavior in the context of people’s real lives, generating a rich data set that can provide insights into the extent and timing of mental health needs in individuals [6].

However, long-standing problems have hampered the efforts to improve mental health care delivery, quality of care, and social support. For example, if mental health conditions are assessed exclusively on patients’ self-reporting, it may be burdensome to collect and subjective for clinical decision support. Currently, mental health services are mainly provided at times chosen by the practitioner rather than at the patient’s time of greatest need [7]. The ideal way of providing support is to conduct regular assessments, which is useful for capturing the temporal dynamics of symptoms and crucial for both diagnosis and treatment planning [8]. However, this could contribute to burnout among health care providers and patients [9].

The emerging health technologies and digital services provide effective ways of collecting human behavior information, gathering patient-generated health data (PGHD), and sharing health-related information outside clinical settings in a systematic way, thus making interventions timely. Coupled with population health informatics tools, these technologies can track people’s digital exhaust, which includes PGHD and social networking platform use. Social networking services are web-based platforms that people use to build social networks or social relationships with other people who share similar personal interests, activities, backgrounds, or real-life connections [10]. The rich real-time data enable researchers to gain insights into aspects of behavior that are well-established building blocks of mental health and illness, such as mood, social communication, sleep, alcohol use, and physical activity.

### Objectives

**Figure 1:**
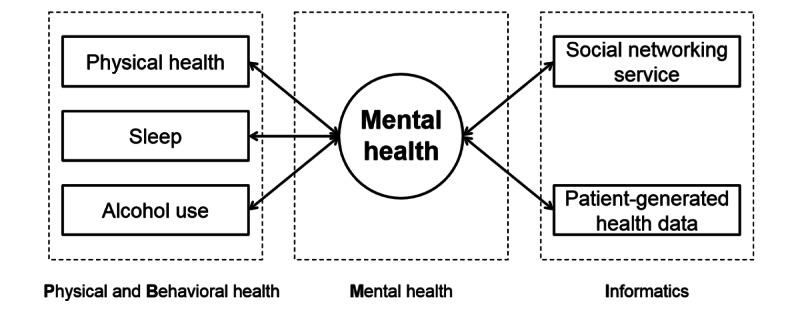
Physical and Behavioral Health, Mental Health, and Informatics (PBMI) framework.

This study had 2 aims. The first aim was to provide a conceptual framework that will be used to describe the relationship between physical and behavioral health, mental health, and informatics. Figure 1 demonstrates the conceptual framework—Physical and Behavioral Health, Mental Health, and Informatics (PBMI)—for this study. The results could provide a comprehensive understanding of the relationship between health, behavior, and informatics, which could be useful for developing tailored and effective strategies to support mental health management. The second aim was to describe and compare characteristics of populations with and without mental health issues (depression or anxiety disorders), including physical health, sleep, and alcohol use, based on the proposed PBMI framework. We also examined the patterns of social networking service use, PGHD, and attitudes toward health information sharing and activities.

## Methods

### Study Design

Data for this study were drawn from the 2019 Health Information National Trends Survey (HINTS) of the National Cancer Institute. HINTS is a nationally representative survey administered every year by the National Cancer Institute that provides a comprehensive assessment of the American public’s current access to and use of health information [11]. The HINTS target population is civilian, noninstitutionalized adults aged ≥18 years living in the United States. In this study, we investigated the relationships between mental health, physical health, behavioral health, and social networking service use. Social networking services include sharing health information, writing online diary blogs, participating in online forums or health-related groups, and watching health-related videos.

### Study Participants

The data used in this study were from the third round of data collection for HINTS 5 (cycle 3), which was conducted from January 22, 2019, to April 30, 2019. Cycle 3 received 5590 questionnaires, of which 5438 (97.28%) were determined to be eligible after excluding blank, incomplete, and duplicate surveys.

In this study, the primary outcome was the presence of mental health issues, which was determined by the participant’s status of depression or anxiety disorder based on the results of the question *Has a doctor or other health professional ever told you that you had depression or anxiety disorder (yes/no)?* Of the 5438 eligible respondents, 1139 (20.95%) reported *yes*, 4168 (76.65%) reported *no*, and 131 (2.41%) were missing and omitted from our analyses.

### Measures

#### Social Determinants of Health

The sample was divided into 2 groups according to mental health status. Participants with depression or anxiety disorders were classified as the group with mental health issues, and the others were classified as the group with no mental health issues. We used the participants’ self-reported information on age, sex, race, ethnicity, level of education, annual income, and usual source of care as our sociodemographic variables. We transformed the continuous variable of age into a categorical variable by classifying age into four groups: (1) 18 to 34 years, (2) 35 to 49 years, (3) 50 to 64 years, and (4) ≥65 years. Education level was recategorized as less than college (including post–high school training), some college, college graduate, and postgraduate degree. Annual income level was recategorized as ≤US $20,000, US $20,000 to $35,000, US $35,000 to $50,000, US $50,000 to $75,000, and >US $75,000. We examined the participants’ history of chronic conditions using four questions (all with yes or no responses): *Has a doctor or other health professionals ever told you that you had (1) diabetes, (2) high blood pressure, (3) a heart condition, and (4) chronic lung disease?*

#### Health-Related Information

To assess the participants’ general health, we considered their answers to questions related to physical health and mental status, including the ability to take care of their health, emotion control by changing the way of thinking, and future consideration. We also included the Patient Health Questionnaire–4 (PHQ-4), which was a derived composite from the participants’ responses to questions on lack of interest in doing things, presence of depressed feelings, nervousness and anxiousness, and uncontrolled worry [12].

#### PGHD and Social Networking Service Use

We examined the participants’ use of the internet for health-related reasons using the following five survey questions (all with yes or no responses): *In the past 12 months, have you used the internet to (1) visit a social networking site, such as Facebook or LinkedIn, (2) share health information on social networking sites, such as Facebook or Twitter, (3) write in an online diary or blog, (4) participate in an online forum or support group for people with similar health or medical issue, or (5) watch a health-related video on YouTube?*

We also inspected the first source of health information of the participants using their responses to the following question—*The most recent time you looked for information about health or medical topics, where did you go first?*—where the respondent could select one of 12 options. We further grouped the options into five main categories: internet, health professionals, family and friends, print materials, and others. In addition, we investigated the participants’ attitudes toward sharing health information, such as avoidance of physician visits and talking about health with family and friends.

#### Alcohol Consumption and Sleep

We examined the participants’ alcohol consumption using two questions: the number of days with at least one alcoholic drink per week and the average number of drinks per day. We assessed the participants’ sleep hours and quality using two questions: the average number of hours of sleep per night and the self-rated overall sleep quality. We transformed the continuous variable of average sleep per night into a categorical variable by classifying sleep hours as (1) 0 to 6 hours, (2) 7 to 8 hours, and (3) ≥9 hours.

### Statistical Analysis

We used the survey package in the R programming language (version 4.0.5; R Foundation for Statistical Computing) to account for the complex sampling design used in HINTS and incorporated the Taylor series (linear approximation) [13] to generate accurate variance estimation. All analyses used weighted data based on the Taylor series method to calculate population estimates. Pairwise deletion was used to deal with missing data to preserve more information.

To assess sociodemographic characteristics, general health, chronic diseases, social networking service use, alcohol consumption, and sleeping variables, we generated weighted 2-way cross-tabulation tables, which were tested with a Pearson chi-square test of association.

A univariate logistic regression was built to examine the association between each predictor and mental health. We then performed multivariate logistic regression analyses using a survey-weighted generalized linear modeling function in R [14]. The variables included the participants’ sociodemographic and clinical characteristics. Odds ratios (ORs) and 95% CIs for both models were calculated. All reported *P* values were 2-tailed, and a cutoff of *P*<.05 was used to determine statistical significance for all analyses.

### Ethics Approval

The data for this study are publicly available.

## Results

### Population Characteristics

Table 1 reports the sociodemographic and clinical characteristics of the participants. Respondents with mental health issues were significantly more likely to be younger (*P*=.004), White (*P*=.005), and female (*P*<.001); have a lower income (*P*<.001); and have a usual source of care (*P*<.001). They were also more likely to have a history of diabetes (*P*=.001) and lung disease (*P*<.001). There were no significant differences between the 2 groups regarding the characteristics of history of hypertension (*P*=.10), heart condition (*P*=.40), and cancer (*P*=.13).

**Table 1:** Unweighted and weighted prevalence estimates for sample sociodemographic characteristics, Health Information National Trends Survey 5, cycle 3.

Characteristic			Overall (n=5438), unweighted %		Overall (n=252,070,495), weighted %		Having mental health issues (n=57,953,433), weighted %		No mental health issues (n=189,456,090), weighted %		*P* value	
**Age (years)**												.004
	18 to 34	13		24.3		28.1		23.1				
	35 to 49	18.3		24.5		26.3		24.2				
	50 to 64	31.6		31.1		32.8		30.7				
	≥65	37.1		20.2		12.8		22				
Sex (male)			42.1		48.8		37.9		52.5		<.001	
**Race^a^**												.005
	White	73.9		77		82.9		75				
	Black	16.5		13		9.9		13.9				
	Asian	5		5.8		3.4		6.7				
	Others	4.7		4.2		3.9		4.4				
Ethnicity (Hispanic)			14.9		16.7		13.9		17.6		.07	
**Education**												.41
	High school diploma or less	54.4		70.5		72.5		69.7				
	College degree	26.5		17.3		16.1		17.8				
	Postgraduate degree	19.1		12.2		11.4		12.5				
**Income (US $)**												<.001
	<20,000	18.8		18.5		26.2		15.6				
	20,000 to 34,999	12.8		11		11.6		10.7				
	35,000 to 49,999	13.1		13.5		14.2		13.4				
	50,000 to 74,999	17.7		17.4		16.8		17.7				
	≥75,000	37.6		39.6		31.3		42.6				
**Insurance**												.18
	Public	44.1		35.2		38.9		33.5				
	Private	42.3		48.9		44.8		50.8				
	Uninsured	4.8		7.7		7.2		7.8				
	Others^b^	8.8		8.2		9.2		8				
Has a usual source of care			69.8		64.5		73.7		61.7		<.001	
History of cancer			16.1		9.5		7.9		9.7		.13	
History of lung disease			11.8		11.2		2		8.2		<.001	
History of heart condition			16.1		8.1		9.2		7.8		.40	
History of diabetes			21.7		17		22.4		15.2		.001	
History of hypertension			45		36		39.1		34.9		.10	

^a^Asian Indian, Chinese, Filipino, Japanese, Korean, Vietnamese, and other Asian were collapsed into the *Asian* category. Race categories other than White, Black, and Asian were reclassified as *Others*.

^b^*Others* include coverage under the spouse, coverage under parents, and low-income beneficiary.

### Health Information and Social Networking Service

Table 2 shows the characteristics of health information source, health information sharing, and social networking service use. Participants with mental health issues were more likely to have a worse general health status (*P*<.001), less confidence in taking care of their own health (*P*<.001), and a higher PHQ-4 score (*P*<.001). Individuals with mental health issues were also less likely to control emotions by changing the way they thought about situations (*P*<.001) and try to influence things in the future with day-to-day behavior (*P*<.001). In addition, Table 2 shows that those with mental health issues were significantly more likely to visit social networking sites (*P*=.04), share health information on social networking sites (*P*=.001), write online diary blogs (*P*=.007), participate in an online forum or support group (*P*<.001), and watch health-related videos (*P*=.009). There were no significant differences between the 2 groups in the first source of health information (*P*=.23), using wellness apps (*P*=.33), avoidance of physician visits (*P*=.15), and talking about health with family or friends (*P*=.08). Multimedia Appendix 1 shows the results of the multivariate logistic regression of health information and social networking service use.

**Table 2:** Prevalence estimates for characteristics of health information and social networking service use.

Characteristic			Overall (n=5438), unweighted %		Overall (n=252,070,495), weighted %		Having mental health issues (n=57,953,433), weighted %		No mental health issues (n=189,456,090), weighted %		*P* value	
**Source of health information**												.23
	Internet	42.9		46.1		50.2		45.2				
	Health professionals	48.9		44.6		39.9		45.9				
	Family or friends	4.1		5.2		4.6		5.3				
	Print materials	2.3		2.2		2.4		2.1				
	Others	1.8		2		2.9		1.5				
**Use of health apps**												.33
	Yes	52.4		54.8		57.7		54.2				
	No	42.2		39.6		35.9		40.6				
	Do not know	5.4		5.5		6.4		5.2				
Good health status			47.9		49.4		33.7		54.2		<.001	
Have ability to take care of health			72.2		71.5		56		76.3		<.001	
Avoid visiting physician			25		30.6		33.9		29.6		.15	
Talks about health with family or friends			81		78.5		81.9		77.4		.08	
**PHQ**-**4^a^**												<.001
	0	50.3		45.6		13.6		54.9				
	1	12.3		13.2		9.3		14.4				
	2	9.9		9.6		11.5		9.1				
	≥3	27.6		31.6		65.5		21.6				
Can control emotions			85.1		84.5		77.6		86.7		<.001	
Consider future			84.5		84.7		79.2		86.6		<.001	
Visit social networking sites			65		71.5		76		70.8		.04	
Share health information			11.9		14.6		19.7		13.1		.001	
Write online diary blog			3.6		5		8		4		.007	
Participate in online forum or health-related group			7		8.1		13.3		6.6		<.001	
Watch health-related videos			32.8		37.3		42.5		35.9		.009	

^a^PHQ-4: Patient Health Questionnaire–4.

### Alcohol Consumption and Sleep

Table 3 shows the characteristics of behavioral health, including sleep and alcohol use, of the 2 groups. Individuals with mental health issues were more likely to sleep <6 hours or >9 hours (*P*=.01), have worse sleep quality (*P*<.001), and consume more alcohol per day (*P*=.03). There was no significant difference in the number of days of alcohol consumption per week between the 2 groups.

**Figure 2:**
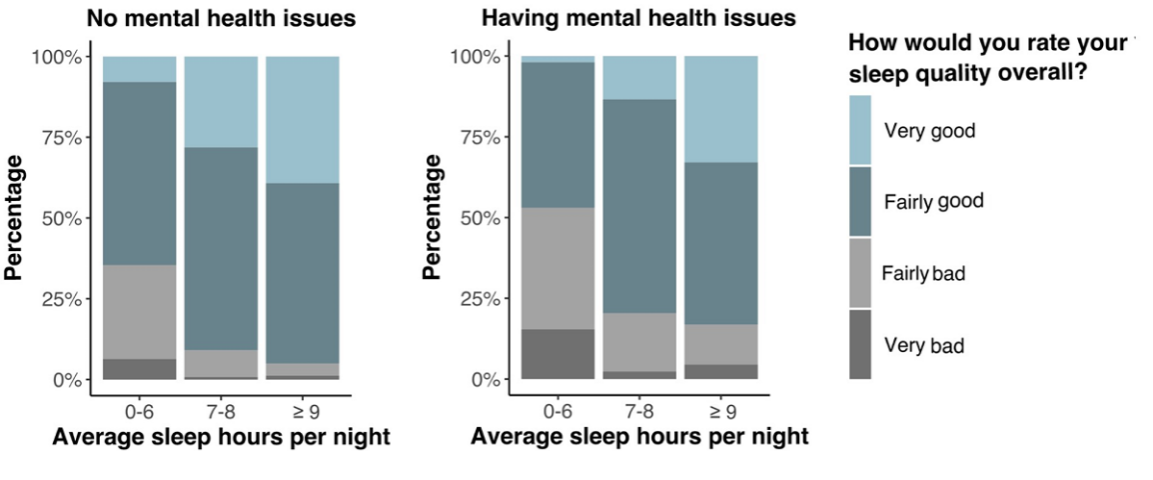
Sleep patterns between the 2 mental health groups.

Figure 2 illustrates the difference in sleep quality among individuals who slept ≤6 hours, 7 to 8 hours, and >9 hours per night between the 2 groups. For individuals with mental health issues, 52% of those who slept ≤6 hours per night had a poor sleep quality. Among individuals without mental health issues, only 9.1% of those who slept 7 to 8 hours per night had a poor sleep quality, which is significantly less than that of individuals with mental health issues who slept the same hours.

We examined whether sleep quality was the same for populations with and without mental health issues separately within the 3 sleep hour categories (0-6 hours, 7-8 hours, and ≥9 hours). As the normality assumption is unjustified, we conducted the Mann-Whitney *U* test. For people who slept 0 to 6 hours (*P*<.001) and 7 to 8 hours (*P*<.001), there was a significant difference in sleep quality between the 2 groups. There was no significant difference in sleep quality for people who slept >9 hours (*P*=.14) between the 2 groups. We found that individuals without mental health issues slept 7 to 8 hours with higher quality, whereas patients with mental health issues slept <6 hours or >9 hours with poor quality.

**Figure 3:**
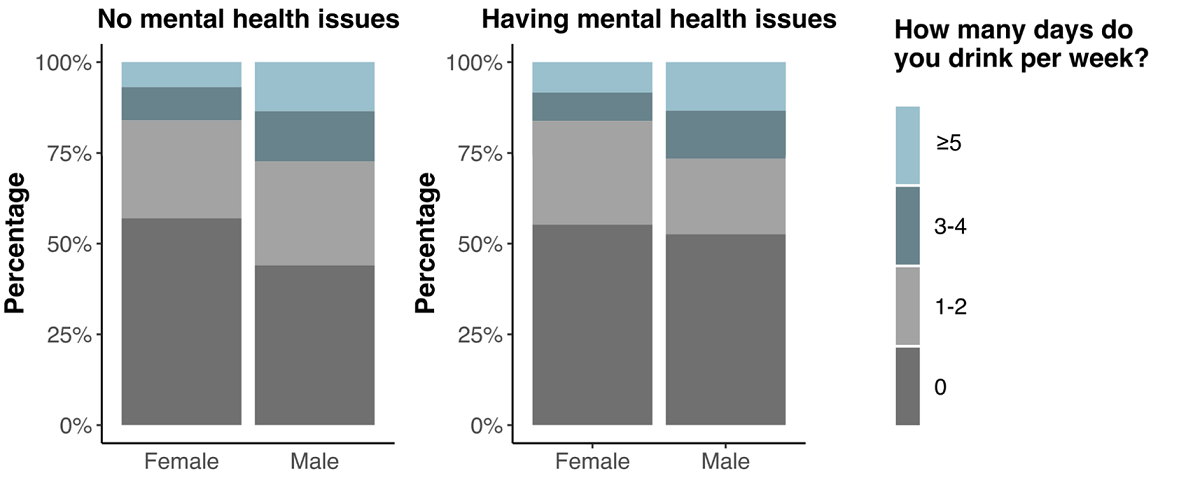
Alcohol use patterns stratified by sex and mental health status.

Figure 3 illustrates the difference in the amount of alcohol consumed per week between the groups stratified by sex and mental health status. Approximately 43% (38,696,406/89,991,643) of women without mental health issues consumed one or more drinks per week, whereas 44.8% (16,123,109/35,989,082) of women with mental health issues consumed the same number of drinks per week. We found no significant difference in drink amount between women (*P*=.66) and men (*P*=.23) regardless of mental health status. However, for individuals without mental health issues, men drank significantly more than women (*P*<.001).

**Table 3:** Prevalence estimates for characteristics of sleep and alcohol use.

Characteristic			Overall (n=5438), unweighted %		Overall (n=252,070,495), weighted %		Having mental health issues (n=57,953,433), weighted %		No mental health issues (n=189,456,090), weighted %		*P* value	
**Sleep hours per night**												.01
	0 to 6	38.9		38.9		41.4		38.2				
	7 to 8	53.3		54.1		49		55.7				
	≥9	7.8		7		9.6		6.1				
**Overall sleep quality**												<.001
	Very good	20		18.8		10.5		21.1				
	Fairly good	58.2		58.7		55.7		59.8				
	Fairly bad	17.8		18.3		25.7		16.1				
	Very bad	4		4.2		8		3				
**Days consuming alcohol per week**												.52
	0	50.5		51.3		54.2		50.3				
	1 to 2	26.5		27.3		25.6		27.9				
	3 to 4	10.8		11.1		9.8		11.5				
	≥5	12.2		10.3		10.5		10.3				
**Alcohol drinks per day**												.03
	0 to 1	43.2		37.7		29.3		40.1				
	2 to 3	43.9		45.3		51.4		43.6				
	≥4	12.9		17		19.3		16.4				

### Social Determinants of Health and Mental Health

Table 4 shows the results of logistic regression analyses. In the unadjusted logistic regression model, most covariates were associated with mental health. In the adjusted model, those aged ≥65 years had a reduced likelihood (OR 0.20, 95% CI 0.11-0.35) of having mental health issues compared with those aged 18 to 34 years. Men were less likely (OR 0.52, 95% CI 0.40-0.68) to have mental health issues. The Black population had a reduced likelihood (OR 0.41, 95% CI 0.27-0.63) of having mental health issues compared with the White population.

Individuals who had an annual family income <US $20,000 were more likely (OR 2.39, 95% CI 1.48-3.84) to have mental health issues than those whose income was >US $75,000. Those having a usual source of care were more likely (OR 1.72, 95% CI 1.24-2.39) to have mental health issues. As expected, those with a history of lung disease (OR 2.17, 95% CI 1.54-3.05), diabetes (OR 1.42, 95% CI 1.03-1.95), and hypertension (OR 1.40, 95% CI 1.07-1.84) were more likely to have mental health issues. The results also indicated that ethnicity, education, insurance type, history of cancer, and history of heart condition had no association with mental health status.

**Table 4:** Crude and adjusted odds from logistic regression analyses of associations between social determinants of health and mental health.

Predictor		Unadjusted			Adjusted	
		OR^a^ (95% CI)	*P* value	OR (95% CI)		*P* value
**Age (years)**						
	18 to 34	Reference	Reference	Reference		Reference
	35 to 49	0.89 (0.61-1.31)	.56	0.99 (0.64-1.54)		.98
	50 to 64	0.88 (0.60-1.29)	.51	0.60 (0.38-0.96)		.04
	≥65	0.48 (0.32-0.71)	<.001	0.20 (0.11-0.35)		<.001
Sex (male)		0.55 (0.43-0.70)	<.001	0.52 (0.40-0.68)		<.001
**Race**						
	White	Reference	Reference	Reference		Reference
	Asian	0.46 (0.25-0.86)	.02	0.51 (0.25-1.03)		.06
	Black	0.64 (0.46-0.90)	.01	0.41 (0.27-0.63)		<.001
	Others	0.79 (0.48-1.32)	.37	0.52 (0.25-1.08)		.08
Ethnicity (Hispanic)		0.76 (0.56-1.02)	.07	0.73 (0.48-1.11)		.14
**Education**						
	High school diploma or less	Reference	Reference	Reference		Reference
	College degree	0.87 (0.68-1.12)	.28	0.91 (0.64-1.31)		.63
	Postgraduate degree	0.87 (0.65-1.17)	.37	0.92 (0.59-1.42)		.69
**Income (US $)**						
	<20,000	2.29 (1.65-3.17)	<.001	2.39 (1.48-3.84)		<.001
	20,000 to 34,999	1.48 (1.00-2.19)	.05	1.59 (1.00-2.55)		.05
	35,000 to 49,999	1.44 (0.97-2.14)	.07	1.34 (0.83-2.15)		.23
	50,000 to 74,999	1.29 (0.89-1.86)	.18	1.32 (0.88-1.98)		.19
	≥75,000	Reference	Reference	Reference		Reference
**Insurance**						
	Public	Reference	Reference	Reference		Reference
	Private	0.76 (0.59-0.98)	.03	0.71 (0.47-1.07)		.11
	Uninsured	0.79 (0.48-1.31)	.37	0.56 (0.29-1.09)		.09
	Others	0.99 (0.65-1.51)	.98	0.94 (0.59-1.51)		.81
Has a usual source of care		1.74 (1.36-2.24)	<.001	1.72 (1.24-2.39)		.001
History of cancer		0.81 (0.61-1.07)	.14	0.88 (0.61-1.28)		.51
History of lung disease		2.99 (2.21-4.04)	<.001	2.17 (1.54-3.05)		<.001
History of heart condition		1.19 (0.79-1.80)	.40	0.94 (0.60-1.47)		.79
History of diabetes		1.61 (1.23-2.10)	<.001	1.42 (1.03-1.95)		.03
History of hypertension		1.20 (0.97-1.48)	.10	1.40 (1.07-1.84)		.01

^a^OR: odds ratio.

## Discussion

### Principal Findings

This study aimed to describe and compare the characteristics of populations with and without mental health issues (depression or anxiety disorders), including physical health, sleep, and alcohol use. We examined the patterns of social networking service use, PGHD, and attitudes toward health information sharing and activities. We found that participants who were younger, White, and female; had a lower income; had a history of chronic disease; and had a higher PHQ-4 score were more likely to have mental health problems, which is consistent with previous findings [15]. Overall, social determinants of health such as age, race, income, insurance status, and chronic diseases, including lung disease, diabetes, and hypertension, were associated with mental health. Participants with mental illness were more likely to visit social networking sites, share health information on social networking sites, write online diary blogs, participate in online forums or support groups, and watch health-related videos. We also found that participants with mental illness slept less with worse sleep quality and consumed more alcohol per day.

Health disparities exist between women and men and among different races with regard to mental health. Mental health issues result in less sleep with poor quality and unhealthy alcohol consumption behaviors. Individuals with mental health issues are more likely to use social networking platforms, share their health information, and actively engage in PGHD. The results provide important insights into the interplay between three vital health-related domains—physical health, behavioral health (sleep and alcohol use), and social networking service use and their patterns in populations with mental health issues.

In recent years, there has been increasing acknowledgment of the important role that mental health plays in achieving improved population health. Understanding how these fundamental factors (physical and behavioral health, mental health, and technologies) relate to one another may yield important insights for novel approaches to designing prevention programs and enhancing services for mental health support. Digital health technologies such as smartphone apps and social media provide opportunities to continuously collect objective information on behavior in the context of people’s real lives, generating a rich data set that can provide insights into the extent and timing of mental and physical health needs in individuals [6].

### Social Networking Service

Individuals who have depression and anxiety are more likely to use social networking platforms, especially younger people. They also tend to be less likely to control emotions by changing the way they think about situations and try to influence things in the future with day-to-day behavior. Social networking plays an important role for this population to find ways to reduce loneliness or symptoms of mental health problems.

We also found that women had a higher level of vulnerability to poor mental health compared with men, which aligned with previous findings [16]. There is an ongoing debate on whether the use of mobile health technologies such as social media is detrimental to mental health [17]. Interestingly, those with depression or anxiety disorders were significantly more likely to visit social networking sites, write online diary blogs, participate in an online forum or support group, and watch health-related videos. These social networking platforms could potentially provide effective strategies to intervene in mental illness. We acknowledge that safe limited use of social media is beneficial, but it could introduce harmful influences if people spend too much time in this digital and internet-based world [18]. Further research is needed to understand the quantitative and dynamic patterns of social media use to measure its benefits and harmful effects and inform evidence-based approaches to clinical interventions, practices, policy, education, and regulation [19]. If we take advantage of the social networking services and data-gathering functions of digital platforms in the right ways, we may achieve breakthroughs in the technologies’ ability to support mental health and well-being.

### Mental Health and PGHD

This study found that individuals with depression or anxiety disorders were willing to share health information on social networking sites, which offers an opportunity to provide interventions that are timely, personalized, and scalable. Coupled with telehealth or remote management platforms [20], practitioners could provide mental health services and support in a timelier manner and at each individual’s time of greatest need. Digital health platforms and PGHD are facilitating the development of a wave of timely interventions for mental health care and support [7]. Big data technologies are facilitating the integration of PGHD and electronic health records, which will encourage the use of predictive analytics and artificial intelligence such as natural language processing and machine learning on structured and unstructured data to help health care providers, hospitals, and patients make their data more meaningful. These findings may be useful for stakeholders such as health care providers, researchers, public health practitioners, and mobile health and social media companies and encourage them to work jointly to design and provide *precision social networking service* with higher personalized and participatory levels, thus improving population health.

### Mental Health, Alcohol Use, and Sleep

This study found that individuals without mental health issues slept 7 to 8 hours with higher quality, whereas patients with mental health issues slept <6 hours or >9 hours with poor quality. Scientific guidelines for sleep suggest that ≥7 hours of sleep per night are appropriate for adults aged 18 to 60 years, 7 to 9 hours are appropriate for adults aged 61 to 64 years, and 7 to 8 hours are appropriate for adults aged ≥65 years [21,22]. Although the amount of sleep is important, other aspects of sleep also contribute to health and well-being. Good sleep quality is also essential. We found that patients with mental health issues were more likely to sleep too much, which is not recommended by health professionals. Previous studies have shown that adolescents and young adults are prone to both mental health and sleep problems [23]. Sleep quality may be particularly important for young adults such as college students with poor mental health who, compared with their peers, tend to lack protective social support networks [24].

Among individuals who are already susceptible to alcohol use, inadequate sleep may further weaken their cognitive capacity to make safer drinking-related decisions or their self-protective behaviors irrespective of consumption levels. Further investigations are needed to examine how poor mental health relates to both alcohol consumption and consequences as well as the extent to which alcohol consumption may mediate the relationship between mental health and consequences. Digital social platforms play a vital role in educating people on alternative coping or harm-reduction skills to use in drinking contexts.

Given the important role of different types of drinking motives in the connection between mental health and drinking outcomes, it is important to examine drinking motivations as mediators of this relationship [25]. Furthermore, event-level methods that simultaneously account for individuals’ sleep and alcohol use behaviors may be helpful for future longitudinal research.

### Limitations

The sample consisted of missing data regarding health outcomes and covariates, which may not be missing completely at random. Those who did not respond to questions may be less active and, thus, our estimates may be subject to bias. As the survey was cross-sectional, we could not examine causality among the variables. Meanwhile, given the limitations of the data set, we did not have information about the use frequency and duration of social networking platforms. Despite these limitations, this study provides a better understanding of the effects and patterns of social networking service use, PGHD, social determinants of health, and mental health.

### Conclusions

This study provided a conceptual framework—PBMI—that could be used to describe the relationship between physical and behavioral health, mental health, and informatics. With this framework, we described the health disparities that existed between women and men and among individuals of different races with regard to mental health, patterns of using social networking platforms, sharing health information, and engagement in PGHD. Leveraging digital platforms and population informatics such as mobile health and social media along with PGHD could offer unique opportunities to develop effective self-monitoring and self-management strategies for supporting patients with mental health issues.

## Appendix

Multimedia Appendix 1 Multivariable logistic regression of health information and social networking service use.

## Abbreviations

HINTS: Health Information National Trends Survey
OR: odds ratio
PBMI: Physical and Behavioral Health, Mental Health, and Informatics
PGHD: patient-generated health data
PHQ-4: Patient Health Questionnaire–4
