# Correction: Social Networking Service, Patient-Generated Health Data, and Population Health Informatics: National Cross-sectional Study of Patterns and Implications of Leveraging Digital Technologies to Support Mental Health and Well-being

**DOI:** 10.2196/84389

**Published:** 2025-10-08

Following the publication of “Social Networking Service, Patient-Generated Health Data, and Population Health Informatics: National Cross-sectional Study of Patterns and Implications of Leveraging Digital Technologies to Support Mental Health and Well-being” [1], concerns were raised regarding the corresponding author Jiancheng Ye’s rate of self-citation within the article’s reference list. Further investigation into this matter identified that numerous self-citations were added to the manuscript following acceptance of the article without adequate disclosure.

All authors were contacted for a response regarding this matter. All authors indicated that they approved the final version of the manuscript and that they believed the added references were pertinent to the article and its findings.

Following review of these responses, the JMIR Publications Editorial Office determined that several of the added references were not relevant to the article or were used to support a generic statement where another reference could have been used instead of self-citation. Therefore, the JMIR Publications Editorial Office is issuing this corrigendum to remove the following references:

- Reference 10: Ye J. The role of health technology and informatics in a global public health emergency: practices and implications from the COVID-19 pandemic. JMIR Med Inform 2020 Jul 14;8(7):e19866 [doi: 10.2196/19866] [Medline: 32568725]
- Reference 15: Ye J, Ma Q. The effects and patterns among mobile health, social determinants, and physical activity: a nationally representative cross-sectional study. AMIA Jt Summits Transl Sci Proc 2021 May 17;2021:653-662 [Medline: 34457181]
- Reference 22: Ye J, Li N, Lu Y, Cheng J, Xu Y. A portable urine analyzer based on colorimetric detection. Anal Methods 2017;9(16):2464-2471 [doi: 10.1039/c7ay00780a]
- Reference 24: Ye J. Health information system’s responses to COVID-19 pandemic in China: a national cross-sectional study. Appl Clin Inform 2021 Mar 19;12(2):399-406 [doi: 10.1055/s-0041-1728770] [Medline: 34010976]
- Reference 25: Ye J, Yao L, Shen J, Janarthanam R, Luo Y. Predicting mortality in critically ill patients with diabetes using machine learning and clinical notes. BMC Med Inform Decis Mak 2020 Dec 30;20(Suppl 11):295 [doi: 10.1186/s12911-020-01318-4] [Medline: 33380338]
- Reference 26: Ye J, Sanchez-Pinto LN. Three data-driven phenotypes of multiple organ dysfunction syndrome preserved from early childhood to middle adulthood. AMIA Annu Symp Proc 2021 Jan 25;2020:1345-1353 [Medline: 33936511]
- Reference 27: Ye J. Advancing mental health and psychological support for health care workers using digital technologies and platforms. JMIR Form Res 2021 Jun 30;5(6):e22075 [doi: 10.2196/22075] [Medline: 34106874]

The JMIR Publications Editorial Office regrets that these issues were not identified prior to publication.

The correction will appear in the online version of the paper on the JMIR Publications website together with the publication of this correction notice. Because this was made after submission to PubMed, PubMed Central, and other full-text repositories, the corrected article has also been resubmitted to those repositories.
