# Supplementary material for: Social Networking Service, Patient-Generated Health Data, and Population Health Informatics: National Cross-sectional Study of Patterns and Implications of Leveraging Digital Technologies to Support Mental Health and Well-being
Source: J Med Internet Res. 2022 Apr 29;24(4):e30898. doi: 10.2196/30898 (PMC9107051; doi:10.2196/30898)
Supplement: Multimedia Appendix 1 [file jmir_v24i4e30898_app1.docx]

Supplemental table 1. The multivariable logistic regression of health information and social networking service

|  | **Unadjusted** | | **Adjusted*** | |
| --- | --- | --- | --- | --- |
| **Predictor** | **OR (95% CI)** | **P value** | **OR (95% CI)** | **P value** |
| **Source of health information** |  |  |  |  |
| Internet | Reference |  | Reference |  |
| Health professionals | 0.79 (0.62, 0.99) | 0.04 | 0.84 (0.65, 1.08) | 0.18 |
| Family or friends | 0.78 (0.41, 1.48) | 0.44 | 0.88 (0.39, 1.97) | 0.75 |
| Print materials | 1.02 (0.49, 2.14) | 0.96 | 1.29 (0.56, 2.97) | 0.55 |
| Others | 1.77 (0.59, 5.27) | 0.30 | 1.93 (0.48, 7.72) | 0.35 |
| **Use health apps** |  |  |  |  |
| Yes | Reference |  | Reference |  |
| No | 0.83 (0.65, 1.06) | 0.14 | 0.94 (0.71, 1.26) | 0.70 |
| Don’t Know | 1.16 (0.61, 2.21) | 0.65 | 1.34 (0.68, 2.66) | 0.40 |
| **Good health status** | 0.43 (0.34, 0.54) | <0.001 | 0.41 (0.31, 0.55) | <0.001 |
| **Have ability to take care of health** | 0.40 (0.31, 0.50) | <0.001 | 0.42 (0.32, 0.54) | <0.001 |
| **Avoid visiting doctor** | 1.22 (0.93, 1.60) | 0.15 | 1.20 (0.87, 1.66) | 0.26 |
| **Talks about health with family or friends** | 1.33 (0.97, 1.82) | 0.08 | 1.24 (0.86, 1.79) | 0.24 |
| **PHQ 4** |  |  |  |  |
| 0 | Reference |  | Reference |  |
| 1 | 2.61 (1.73, 3.94) | <0.001 | 2.32 (1.45, 3.69) | <0.001 |
| 2 | 5.11 (3.57, 7.32) | <0.001 | 4.76 (3.20, 7.08) | <0.001 |
| 3+ | 12.28 (9.03, 16.70) | <0.001 | 9.83 (6.86, 14.08) | <0.001 |
| **Can control emotions** | 0.53 (0.41, 0.67) | <0.001 | 0.53 (0.40, 0.71) | <0.001 |
| **Consider future** | 0.59 (0.46, 0.76) | <0.001 | 0.57 (0.43, 0.77) | <0.001 |
| **Visit social networking sites** | 1.31 (1.01, 1.69) | 0.04 | 1.04 (0.74, 1.46) | 0.83 |
| **Share health information** | 1.62 (1.20, 2.18) | 0.002 | 1.27 (0.88, 1.83) | 0.21 |
| **Write online diary or blog** | 2.09 (1.21, 3.59) | 0.008 | 1.50 (0.76, 2.95) | 0.24 |
| **Participate online forum or health-related group** | 2.15 (1.45, 3.18) | <0.001 | 1.80 (1.14, 2.86) | 0.01 |
| **Watch health-related videos** | 1.32 (1.07, 1.63) | 0.009 | 1.05 (0.79, 1.40) | 0.72 |

*Adjusting participants’ sociodemographic and clinical characteristics

OR = Odds ratio
